# Apparent survival and cost of reproduction for White-lined Tanager (*Tachyphonus rufus*, Thraupidae) in the northern Atlantic Rainforest, Brazil

**DOI:** 10.1371/journal.pone.0185890

**Published:** 2017-10-10

**Authors:** Phoeve Macario, Mauro Pichorim, Paul F. Doherty, Guilherme S. Toledo-Lima, Tonny M. Oliveira-Júnior, Thanyria P. F. Câmara, Shirley Macjane Melo, João Lucas S. Silveira, Juliana C. Araújo, Leonardo F. França

**Affiliations:** 1 Programa de Pós-Graduação em Ecologia, Universidade Federal do Rio Grande do Norte, Natal, Rio Grande do Norte, Brazil; 2 Laboratório de Ornitologia, Departamento de Botânica e Zoologia, Universidade Federal do Rio Grande do Norte, Natal, Rio Grande do Norte, Brazil; 3 Department of Fish, Wildlife, and Conservation Biology, Colorado State University, Fort Collins, Colorado, United States of America; 4 Programa de Pós-Graduação em Ciências Biológicas, Universidade Federal do Rio Grande do Norte, Natal, Rio Grande do Norte, Brazil; 5 Laboratório de Ecologia de Populações Animais, Universidade Federal Rural do Semi-árido, Mossoró, Rio Grande do Norte, Brazil; Oregon State University, UNITED STATES

## Abstract

Understanding latitudinal variation in avian life-history traits has been a focus of many demographic studies around the world. However, we still know little about annual or intra-annual demographic variation within tropical regions or about how factors such as breeding season and precipitation influence demographic rates. In this study, we estimated intra-annual apparent survival of the White-lined Tanager (*Tachyphonus rufus*) using capture-mark-recapture data from northeastern Brazil. We tested whether survival varied seasonally (breeding vs. non-breeding), with rainfall, by age and residence status in our study area. Intra-annual apparent survival was correlated with the reproductive cycle, being lower during the breeding (0.65 ± 0.16 SE) vs. the non-breeding season (0.97 ± 0.05 SE). The annual apparent survival (~0.6) was relatively low for a tropical species. In both years, we observed highest abundance in spring (November, 3.1–3.7 birds/ha) and lowest abundance in autumn-winter periods (May-August, 1.1–1.4 bird/ha). The low survival during the breeding season probably reflects the trade-off between survival and reproduction and the cost of reproduction. Our findings represent an advance in the understanding of the demography of tropical birds because we did not find a predicted high annual apparent survival, and we elucidated some aspects of intra-annual variation in survival. Further exploration of latitudinal variation in demographic traits, especially in diverse, but poorly known habitats is needed to fully vet and develop life history theories.

## Introduction

The effects of latitudinal variation on the life-history strategies of birds have been studied extensively [1–6]. Currently, species are classified along a slow-fast continuum [7–9]. “Fast” species have early maturation, high fecundity, low adult survival, and are generally found in temperate regions, whereas “slow” species, with the opposite traits, tend to occur in the tropics [9, 10]. These patterns have been found in many species and regions, although the connection between theory and data is still far from complete (e.g., [9, 11–15]). We still know little about annual and intra-annual demographic variation in tropical regions and the potential influence of factors such as the breeding cycle, precipitation, and ecological characteristics.

Reproduction is a fundamentally important life history trait, which may influence survival or longevity of adults. During the breeding cycle, activities such as nest construction, territorial defense, production of eggs, and caring for offspring can contribute to mortality of breeding adults through poor nutrition and increased susceptibility to predation and diseases [16–18]. Differences in survival rates between breeding and non-breeding periods help explain the cost of the reproductive process [15, 18] if breeding effort is linked to survival through a cost of reproduction trade-off [19].

Environmental factors also influence demography of year-round resident birds. Effects of annual seasonality, primarily temperature variation, on survival have been studied frequently in temperate regions [20]. While temperature is more stable in tropical zones, rainfall varies considerably. Understanding the effects of rainfall on demographic parameters helps elucidate the mechanisms of population regulation for birds in tropical and temperate regions (e.g., [14, 15, 21]) as well as mechanisms of species persistence or extinction [10, 22]. Rainfall is an important driver of demographic changes both directly, but also indirectly through effects on food resources [14, 23–25]. Seasonal rains have positive effects by improving plants’ productivity and the amount of available food resources, but can also reduce foraging opportunities (mainly for insectivorous species) or nest success during adverse weather [26–28]. The shortage of resources during the dry season can affect survival, population size, and community structure [27, 29].

The few demographic studies of Neotropical birds have focused mainly on sites in Central America (e.g., Costa Rica [30, 31], Panama [32, 33]) and the Greater Antilles (e.g., Puerto Rico [34], Trinidad [35]). In South America, recent avian survival studies have taken place in Ecuador [13], French Guiana [36], and Brazil [37–39]. Knowledge of Neotropical bird demography is still incipient due to avian and habitat diversity in these countries [13]. The White-lined Tanager (*Tachyphonus rufus*, Thraupidae) is a passerine species endemic to the Neotropics, occurring in the Atlantic Forest, Cerrado, Caatinga and Amazon habitats, and is a common species in the northeastern coast of Brazil [40, 41]. This species is a good model for demographic studies in tropical areas because it occurs over a large area, is locally common, and individuals can be assigned age and sex easily in the field [41]. This tanager is predominantly a frugivore and plays an important role in seed dispersal [42].

Our objective was to estimate intra-annual apparent survival and test whether survival varied over the breeding cycle. We also tested for a trade-off between survival and nesting and a survival cost of reproduction by predicting a lower survival during reproduction. We also predicted for positive effects of rainfall, age, and residence status effects on survival. Finally, we estimated population abundance and the influence of time-varying covariates on temporary movements and detection parameters.

## Materials and methods

### Ethics statement

Our use of mist-nets and banding was approved by the Brazilian biodiversity monitoring agency (Institute Chico Mendes for Biodiversity Conservation—ICMBio, Brazilian National Center for Bird Conservation—CEMAVE, permission 3239). We followed standard ethical protocols for wildlife animals. Time in captivity was kept to the minimum, we did no experimentation, and we released all specimens at the same place where they were captured.

### Study area

We conducted our research at the Barreira do Inferno Rocket Launch Center of the Brazilian Air Force (CLBI—Centro de Lançamento Barreira do Inferno) in Parnamirim, Rio Grande do Norte state, Brazil (5°54’S 35°10’W). The area (~1800 ha) is representative of the northern extreme of the Brazilian Atlantic Rainforest ecoregion [43] and has sandy soils, vegetation cover consisting primarily of medium-sized (~3 m) trees and shrubs, and is known locally as “restinga” forest. The study area is protected from impacts such as logging, fire, and hunting. The climate is characterized as hot and humid, with rainy (March to July) and dry (August to February) seasons and an annual average rainfall of 1261 mm [44]. Data from the CLBI weather station for the study period indicated a total rainfall of 1191 mm in 2010, 1748 mm in 2011, and 829 mm in 2012.

### Study design

We used a capture-mark-recapture (CMR) approach with a robust sampling design [45, 46] to estimate demographic parameters within a 300 x 300 m plot with seven horizontal and vertical grid lines 50 m apart (total area ~12 ha considering a peripheral buffer zone of 25 m). Birds were captured with mist-nets (Ecotone 18 x 3 m, mesh 19 mm and five shelves) at the intersections (*n =* 49) of the grid lines. We did not use playback or include resightings in our analysis. All captured birds were marked with aluminum bands provided by the Brazilian National Center for Bird Conservation (CEMAVE/ICMBio). We conducted eight primary capture sessions at three-month intervals between November 2010 and August 2012. During each primary session, four secondary sessions were conducted, each separated by seven days to minimize effects of trap-shy behavior on capture rates. During each secondary session, birds were captured between 05h00min and 10h00min, banded, and released at the capture site. In case of rain, we closed nets and reopened them on subsequent days. We did not have to close nets due to wind. Male birds with uniform black plumage were classified as adults. Adult females were identified by their brown plumage, a whitish spot at the base of the mandible, and no visible commissure mark. We classified individuals with light brown plumage, a yellow commissure mark, and no whitish spot at the base of the mandible as juveniles [41].

### Data analysis

We assessed population closure in each primary session with program CLOSETEST [47, 48]. We used a Huggins robust design model [46, 49, 50] in program MARK [51] to estimate apparent survival (Φ), temporary movements (*γ’* and *γ”*), capture and recapture probabilities (*p* and *c*) and abundance (N). Based on the presence of brood patches on captured birds, we defined the breeding season as between February and May. We used rainfall accumulated during each primary session interval (respectively 325, 320, 590, 55, 51, 168 and 458 mm) and during each primary session (respectively 14, 103, 291, 72, 23, 86, 160 and 51 mm), to examine the influence of precipitation on apparent survival and capture-recapture, respectively.

To avoid bias in our estimates of survival due to the inclusion of transients (i.e., non-resident individuals that disappeared after the first capture) or juveniles, we applied Time-Since-Marking (TSM) models on survival [52]. We used TSM models because populations of frugivorous species with high mobility like the White-lined Tanager probably contain transients [53, 54]. The TSM models allow the partitioning of apparent survival into survival during the first interval after banding (juveniles and/or transients) and apparent survival in subsequent intervals (residents). The notation we used in such models for survival was “Φ_(age+transients)_” which means that survival differed with age (juveniles vs. adults) and with residence status (residents vs. transients).

The gammas, or temporary movement parameters (*γ’* and *γ”*), estimate the probability that an individual is available for capture. The *γ’* parameter estimates the probability of an individual staying off the study area between primary capture sessions, and *γ”*, the probability of an individual leaving between sessions [46, 55]. For all models we considered structures on *γ’* and *γ”* to be constant among primary sessions as we found in preliminary modeling that we did not have enough data or support to consider more complicated movement rates (e.g., Markovian, even flow or random movement) [46].

Our global model was Φ_(age+transients+t)_ *γ’*_(.)_ *γ”*_(.)_ *p*_(t)_ *c*_(t)_, which considered additive effects of age (juveniles vs. adults), residence status (transients vs. residents), and time (t) on survival (Φ), and time effects on capture (*p*) and recapture (*c*). Starting from this global model, we constructed a candidate model set of all additive combinations of these covariates including constant (.) structures for the parameters. In addition, we tested for the influence of the covariates breeding cycle (breed) and precipitation (rain) on apparent survival and detection parameters. These covariates (breed and rain) are time-varying, thus we excluded nonsensical models with both a general time structure and time-varying covariates on the same parameter, following recommendations of White and Burnham [51]. We incorporated model uncertainty into our estimates using model averaging over all models, which produces an average estimate using QAIC_c_ model weights (see below), as recommended by Buckland et al. [56] and Burnham and Anderson [57]. We estimated the probability of annual apparent survival as the product of the primary session survival estimates for each year, and calculated the standard error using the Delta method [58, 59]. In the Huggins robust design models, abundance (N) is a derived parameter and thus we did not model the effects of covariates on it.

We tested for overdispersion of the data using the median *ĉ* procedure in program MARK and estimated the variance inflation factor (*ĉ*), which we used to adjust the Akaike Information Criterion (QAIC_c_) (see Burnham and Anderson [60]). Best-fitting models were those with the smallest QAIC_c_ values. The QAIC_c_*w*_*i*_ (hereafter *w*_*i*_) represents the relative weight of support of each model. The delta QAIC_c_ (ΔQAIC_c_) is the difference between the QAIC_c_ of the best model and the model under consideration. As a rule of thumb, models with a ΔQAICc ≤ 2 explain variation in the data similarly, and they were considered the top-ranking models [60]. Finally, we looked for uninformative parameters in the top-ranking models following Arnold [61].

## Results

We banded 141 individuals (38 males, 43 females, and 60 juveniles) and had 187 recaptures of 134 individuals (S1 Table). We observed brood patches in 59.3% (*n =* 27) of the females captured in February and 36.4% (*n =* 11) in May, and thus assumed this February-May period to correspond to the breeding season. The results of the CLOSETEST analysis indicated population closure in the first seven primary sessions (*P* > 0.05), but some openness in the eighth session (*P* = 0.001). Our global model (Φ_(age+transients+t)_ *γ’*_(.)_ *γ”*_(.)_ *p*_(t)_ *c*_(t)_) had some overdispersion (*ĉ* = 1.56), and we used this value for adjusting metrics and estimates in program MARK. A model indicating apparent survival varied by age, transient status, and breeding season, and detection parameters varied by breeding season and precipitation ranked highest (Φ_(age+transients+breed)_ *γ’*_(.)_ *γ”*_(.)_ *p*_(rain+breed)_ *c*_(rain+breed)_, *w*_*i*_ = 0.35; Table 1). The next three highest ranked models also reflected the influence of the breeding season on apparent survival, and that of precipitation and the breeding season on capture and recapture rates. The top four models had ΔQAIC_c_ < 2 and their accumulated *w*_*i*_ was 0.9. Upon closer examination we found the third and the fourth models “rain” effect on survival was an uninformative parameter (see Arnold [61]). For the third and fourth model, the addition of this covariate did not markedly improve the fit of the models (ΔQAICc < 2). The top nine models had 99% of the weight and a breeding season effect on apparent survival was in all of them.

**Table 1:** Top nine Huggins robust design models for a population of White-lined Tanager (*T*. *rufus*) in northeastern Brazil. These nine represent 99% of the weight in the entire model set. The global model and the constant model (23^rd^ and 61^st^ ranked model in the set respectively) are shown for comparison.

Models	ΔQAIC_c_	*w*_*i*_	ML	*K*	Dev.
Φ_(age+transients+breed)_ *γ’*_(.)_ *γ”*_(.)_ *p*_(rain+breed)_ *c*_(rain+breed)_^a^	0.00	0.35	1.00	10	936.8
Φ_(age+transients+breed)_ *γ’*_(.)_ *γ”*_(.)_ *p = c*_(rain+breed)_	0.77	0.24	0.68	9	939.6
Φ_(age+transients+rain+breed)_ *γ’*_(.)_ *γ”*_(.)_ *p*_(rain+breed)_ *c*_(rain+breed)_	1.33	0.18	0.51	11	936.0
Φ_(age+transients+rain+breed)_ *γ’*_(.)_ *γ”*_(.)_ *p = c*_(rain+breed)_	1.98	0.13	0.37	10	938.7
Φ_(age+transients+breed)_ *γ’*_(.)_ *γ”*_(.)_ *p*_(rain)_ *c*_(rain)_	5.42	0.03	0.07	9	944.3
Φ_(age+transients+breed)_ *γ’*_(.)_ *γ”*_(.)_ *p = c*_(rain)_	5.66	0.03	0.06	8	946.7
Φ_(age+transients+rain+breed)_ *γ’*_(.)_ *γ”*_(.)_ *p*_(rain)_ *c*_(rain)_	7.12	0.01	0.03	10	943.9
Φ_(age+transients+rain+breed)_ *γ’*_(.)_ *γ”*_(.)_ *p = c*_(rain)_	7.37	0.01	0.03	9	946.2
Φ_(age+transients+breed)_ *γ’*_(.)_ *γ”*_(.)_ *p*_(.)_ *c*_(.)_	7.82	0.01	0.02	8	948.8
Φ_(age+transients+t)_ *γ’*_(.)_ *γ”*_(.)_ *p*_(t)_ *c*_(t)_	13.27	0.00	0.00	20	928.0
Φ_(.)_ *γ’*_(.)_ *γ”*_(.)_ *p*_(.)_ *c*_(.)_	37.98	0.00	0.00	5	985.2

Φ = apparent survival, *γ’* = probability of staying off the study area between sessions, *γ”* = probability of leaving between sessions, *p* = capture, *c* = recapture, *K* = number of parameters, *w*_*i*_ = QAIC_c_ weights, ML = Model likelihood, Dev. = Deviance. “age” is juvenile vs. adult, “transients” is the difference between the interval after first capture vs. intervals after subsequent captures, “rain” is the amount of precipitation, “breed” is the breeding vs. non-breeding seasons, and “t” is time.

^a^ QAIC_c_ value of the best model was 958.7.

**Fig 1:**
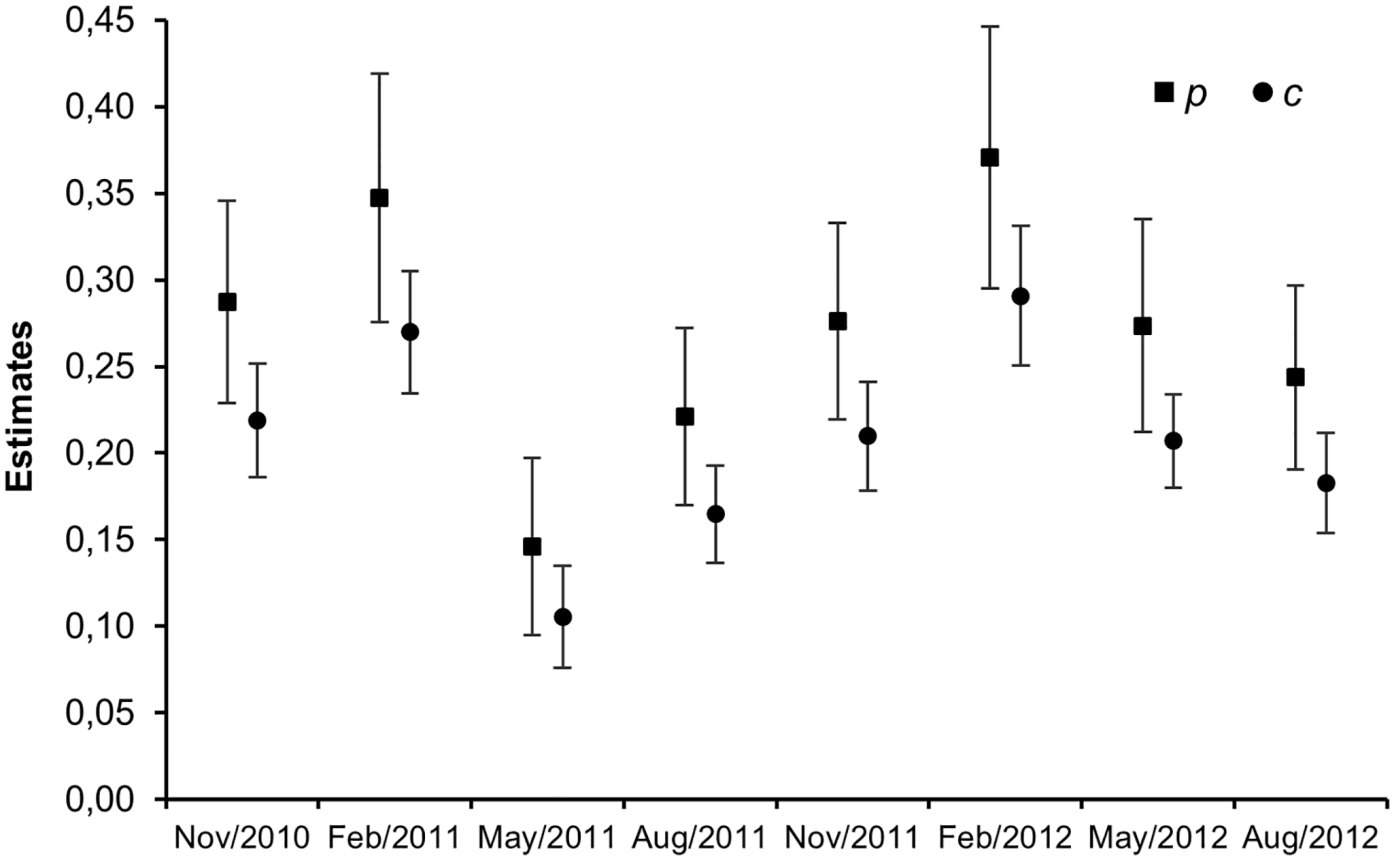
Model-averaged estimates of capture (*p*) and recapture (*c*) based on Huggins robust design models for the White-lined Tanager (*T*. *rufus*) in northeastern Brazil. The vertical bars represent standard error estimates.

The intra-annual apparent survival (survival between the primary three-month session intervals) for resident adults during the non-breeding season was ~1.5 times higher than during the breeding period (February-May, Table 2). Apparent survival of juveniles was ~4 times lower than adult apparent survival during the non-breeding period (Table 2). Because the TSM model structure accounts for transients, our analysis generated six survival estimates for adult residents (Table 2). Based on the first four estimates (February 2011 to January 2012), the annual apparent survival for adult residents was 0.61 ± 0.08. Annual apparent survival estimates from May 2011 to April 2012 and from August 2011 to July 2012, were 0.59 ± 0.08 and 0.58 ± 0.08 respectively. Estimates of detection ranged between 0.10–0.36 for *p* and 0.11–0.29 for *c* (Fig 1). During the primary sessions, the recapture probability was ~1.3 times lower than initial capture probability. In February the capture and recapture probabilities were highest (*p* = 0.33–0.36 and *c* = 0.27–0.29), and lowest in May and August (*p* = 0.14–0.22 and *c* = 0.11–0.17, Fig 1). The probability of entering the study area between primary sessions (1- *γ’* = 0.43) was approximately twice that of leaving (*γ”* = 0.21). The abundance (N) of adults was highest in November 2011 (43.9 ± 8.0) and lowest in May 2012 (13.0 ± 3.7). In both years, we observed a tendency for abundance to be high in November and low in May-August (Table 3).

**Table 2:** Estimates of intra-annual apparent survival based on Huggins robust design models for the White-lined Tanager (*T*. *rufus*) in northeastern Brazil.

Interval	Breeding	Apparent survival (mean ± SE)^a^		
		Transients	Residents	Juveniles
Nov/2010-Feb/2011	No	0.64 ± 0.12	–	0.22 ± 0.09
Feb-May/2011	Yes	0.09 ± 0.10	0.66 ± 0.16	0.02 ± 0.03
May-Aug/2011	No	0.67 ± 0.15	0.98 ± 0.04	0.25 ± 0.09
Aug-Nov/2011	No	0.60 ± 0.13	0.97 ± 0.05	0.20 ± 0.11
Nov/2011-Feb/2012	No	0.60 ± 0.13	0.97 ± 0.05	0.20 ± 0.11
Feb-May/2012	Yes	0.08 ± 0.10	0.64 ± 0.16	0.02 ± 0.03
May-Aug/2012	No	0.65 ± 0.14	0.97 ± 0.04	0.24 ± 0.08

^a^ Apparent survival at three-month intervals between November 2010 and August 2012

**Table 3:** Intra-annual estimates of abundance (mean ± SE) for the White-lined Tanager (*T*. *rufus*) in a 12 ha surveyed area of northeastern Brazil based on Huggins robust design models and conditional on birds being first captured on the study site.

Precipitation (mm)	Session	Breeding	Adults	Juveniles
14	Nov/2010	No	37.4 ± 6.8	Null
103	Feb/2011	Yes	29.2 ± 5.0	3.8 ± 1.4
291	May/2011	Yes	31.4 ± 12.7	92.0 ± 32.0
72	Aug/2011	No	16.3 ± 4.9	13.0 ± 4.3
23	Nov/2011	No	43.9 ± 8.0	14.2 ± 3.6
86	Feb/2012	Yes	35.7 ± 5.5	9.8 ± 2.3
160	May/2012	Yes	13.0 ± 3.7	10.1 ± 3.1
51	Aug/2012	No	15.3 ± 4.3	10.7 ± 3.4

## Discussion

The intra-annual apparent survival of White-lined Tanager in our study area was inversely correlated with the reproductive cycle; apparent survival was considerably lower during the breeding season (~0.65) than the non-breeding period (~0.97). This result may be evidence of a trade-off between survival and reproduction in the White-lined Tanager in our study area. The reduction in apparent survival during the breeding season probably reflects the costs of reproduction, and represents a decrease in the future survival and potential reproduction rates due to present reproductive effort [19]. Previous bird studies have also shown that parental investment in rearing offspring may have a negative effect on adult apparent survival (e.g., [62–64]). Reproductive effort demands energy for the production of eggs, nest defense, feeding nestlings, and increased predator vigilance [16, 19, 65, 66]. The stress of reproductive effort can also leave individuals weaker and more susceptible to disease [16]. In our White-lined Tanager study population the greatest expenditure of energy probably occurred during the construction of nests and the feeding of the nestlings, which occurs between February and May in our study area and which involves both males and females [43]. Sankamethawee et al. [15] obtained a similar result for Puff-throated Bulbul (*Alophoixus pallidus*) in Thailand, albeit with slightly less variation between seasons (0.89 in the breeding season vs. 0.96 in the non-breeding period).

The annual apparent survival we estimated was relatively low, especially for a tropical species. Nevertheless, an annual apparent survival of 60% has been recorded for White-lined Tanager in Trinidad [35], and similar values have been recorded for passerines birds from Ecuador (59% [13]) and Panama (56% [32], 58% [33]). Most other studies of Neotropical species, including passerine and non passerines, have reported much higher estimates (see Table 4). Our estimate was similar to those for some passerines in the temperate zone of North America with indices between 54–60% [32, 67, 68] and considerably higher than estimates (0.44–0.45) recorded for passerines in Europe [69, 70]. Together with the results of previous studies (e.g., [8, 13, 32, 33, 36, 71]), the annual apparent survival we found indicates that not all tropical bird population may have high survival rates [10, 22, 72–74]. In general, tropical birds are known to have low fecundity and high survival, but this generality is based on little information considering the whole biodiversity of the region [9, 10]. Likewise, available data on the survival of Neotropical birds is limited, and biased towards some groups of species and ecosystems, such as understory birds and rainforest areas. Comparative demographic studies at different latitudes should cover regional habitat heterogeneity in the tropics. Until then, reliable generalizations on adult survival rates in natural populations continue to be elusive [75, 76].

**Table 4:** Average annual estimates of apparent survival (Φ; mean ± SD) and the maximum and minimum values estimated for Neotropical birds.

Source	Country	Species (*n*)	Apparent survival (± SD)		
			Mean	Maximum	Minimum
[12]	Ecuador	5	0.57 (± 0.12)	0.86 (± 0.10)	0.56(± 0.11)
[13]	Ecuador	31	0.59 (± 0.02)	0.80 (± 0.15)	0.32 (± 0.09)
[37]	Brazil	1	0.78 (± 0.06)	-	-
[31]	Costa Rica	1	0.80 (± 0.11)	0.97 (± 0.15)	0.74 (± 0.06)
[32]	Panama	25	0.56 (± 0.02)	0.73 (± 0.05)	0.33 (± 0.09)
[33]	Panama	11	0.58 (± 0.03)	0.73 (± 0.04)	0.41 (± 0.07)
[34]	Puerto Rico	9	0.68 (± 0.04)	0.79 (± 0.04)	0.51
[35]	Trinidad	17	0.65 (± 0.10)	0.84 (± 0.05)	0.45 (± 0.07)
[36]	French Guiana	17	0.63 (± 0.16)	0.87 (± 0.05)	0.34 (± 0.07)
[38]	Brazil	1	0.88 (± 0.07)	-	-
[77]	Venezuela	1	0.56	-	-
[78]	Bolivia	1	0.67	-	-
[79]	Ecuador	37	0.60 (± 0.02)	0.80 (± 0.06)	0.42 (± 0.05)

In this context, we note that we can only estimate apparent survival, and we cannot distinguish mortality from permanent emigration. Thus, our estimates are almost certainly lower than true survival [80]. Because we used the robust design and TSM models, our analysis did account for temporary movements and emigration of juveniles and transients, at least partially.

Lower juvenile apparent survival rates (vs. adults, see Table 2) were likely the result of higher mortality and dispersal. In many species, juveniles have high rates of dispersal, which hampers mark-recapture analyses [68]. Fruit is a varied resource with evident seasonality, and young and adults of frugivorous species, such as White-lined Tanager, are more likely to disperse than those of other species [53, 81]. In addition, logistic difficulties often limit the monitoring of individuals during the post-fledging period [82]. Many studies of juvenile survival provide estimates that include losses to both mortality and dispersal, with the latter biasing survival low [68]. Juveniles typically suffer higher natural mortality than adults [22, 83], and post-fledging survival is an important component of the life history affecting persistence of a population [84]. Survival at this life stage is generally lower and more variable than during adulthood [85–87] and is more sensitive to environmental variation [84, 88, 89].

In birds, survival typically increases with age because juveniles are less efficient foragers and are more vulnerable to predation [6, 90, 91]. The difficultly for young tropical birds to acquire foraging skills may lead to low survival rates and may require prolonged behavioral development to attain adult levels of survival [83]. Population modeling that fails to account for differences between juveniles and adults is unlikely to produce accurate predictions of population trends. The inclusion of undistinguished young birds in analyses almost certainly results in underestimates of adult survival rates. Understandably, the most supported models in our study accounted for transients and the age of individuals (TSM models, see Table 1). Based on the results of our study and other researches [12, 32–35, 77], we recommend the application of TSM models in survival analyses, in order to avoid biases derived from the presence of transients and juveniles.

The highest *p* and *c* values in the present study were recorded in February of the breeding season of both study years, and may result from low individual mobility due to territorial defense, nest building, and the feeding of nestlings [19]. The low values observed in the subsequent session (May) may reflect changes in behavior, in particular the cessation of nest building and territorial defense. The estimates of *p* and *c* for May varied considerably between the two years of the study period. We believe that high precipitation during the first quarter of 2011 (456.2 mm) compared with the same period during 2012 (157.2 mm) contributed to a major difference in resource availability, forcing the birds to range more widely in search of food in the second year. In our analysis, the highly ranked models included precipitation as an important explanatory covariate for the detection parameters *p* and *c* (see Table 1).

The observed intra-annual variation in adult population abundance might have been related to the natural population cycle. We saw a tendency for abundance to be higher in November than during May-August (Table 3). November probably marks the beginning of the recruitment of juveniles into the adult population, coinciding with the end of the post-fledgling (pers. obs.). The higher population abundance recorded towards the end of the year may be a response to the recruitment process. Following this period, the population likely stabilizes at lower levels through natural mechanisms of dispersal and the establishment of territories [9, 19].

The low precipitation during the first half of 2012 may have led to the low population abundance observed in May and August of this year. During 2012, the Brazilian Northeast suffered the worst drought in the past four decades [92, 93], and this climatic event probably contributed to the low population densities. For example, in the first half of 2012, we observed a major influx of the migratory Creamy-bellied Thrush (*Turdus amaurochalinus*) at levels never recorded before or since. Between 2010 and 2012, we captured 269 *T*. *amaurochalinus*, of which 203 (75.5%) were trapped in 2012, peaking in May (66 animals captured) and June (62 captures). This thrush is a potential ecological competitor of White-lined Tanager due to the similarities of its foraging behavior [42, 94]. The abundance of many other local passerine species also changed significantly during 2012 (pers. obs.). The increase in the densities of potential competitors in the study area may be one of the reasons for the low densities of White-lined Tanager recorded in 2012 [95–98]. We know that density-dependent effects are more common in sedentary species than migratory ones [99], but we need to better understand how these effects change over time and among regions [100].

We believe that our findings represent an important contribution in the understanding of the demography of a tropical bird, and life-history patterns in our region. Demographic studies of wild bird populations have been conducted for almost a century, although few studies have included the analysis of intra-annual variation. Further demographic studies, primarily in relatively rich, but poorly-known habitats, such as those found throughout much of the tropics, are needed to refine our theories. The role of environmental factors, such as precipitation, temperature, humidity, elevation, and habitat structure, in population dynamics has yet to be fully understood. Ultimately, we will need more knowledge, in particular from experimental studies, to verify the predictions of our models more reliably and conclusively.

## Supporting information

S1 Table Encounter histories. Capture-recapture encounter histories of the White-lined Tanager *Tachyphonus rufus*. It follows a robust design structure and presents 32 occasions (four occasions x eight primary sessions).
(TXT)
